# Re: Urological surgery in the COVID-19 era: Patient counselling and informed consent

**DOI:** 10.1080/2090598X.2020.1788285

**Published:** 2020-07-07

**Authors:** Julien Sarkis, Pierre Sarkis

**Affiliations:** Departments of Urology, Hotel-Dieu de France Hospital, Beirut, Lebanon; Saint Joseph Hospital, Beirut, Lebanon

Dear Editor,

The author [1] exposed an overlooked issue that every urologist is facing during this critical period. Patient’s fear of contracting severe acute respiratory syndrome coronavirus 2 (SARS-CoV-2), as well as the difficulty in engaging with the healthcare system is disrupting the spectrum of medical care services. Consequently, and as stated by the author [1], the urologist’s ‘clinical decision and service provision may need to deviate from the internationally accepted standard of care during this unprecedented situation’. Because the standard of care cannot be always guaranteed, careful patient counselling and consent are a must. In this context, we would like emphasise the delicate situation of patients with cancer, especially those with urological malignancies. This is indeed a high-risk population, known to be extremely susceptible to the infectious complications of SARS-CoV-2 [2]. They are also the ones in most need of life-saving surgeries. But due to operating room closures and saturation of intensive care unit beds, patients with cancer are seeing their surgery modified, delayed or even cancelled during this pandemic [3]. While the author presented pertinent solutions (e.g. following international society guidelines) to protect these patients, we cannot stress enough the importance of maintaining traceable multidisciplinary team discussions (MDTs). The latter can propose evidence-based alternatives for surgeries, without compromising prognosis. Recently, the USA Food and Drug Administration (FDA)-approved pembrolizumab, and this for example, could be considered for patients with BCG-unresponsive, high-risk, non-muscle-invasive bladder cancer with carcinoma *in situ* who cannot undergo cystectomy (Phase II KEYNOTE-057 trial, ClinicalTrials.gov Identifier: NCT02625961) [4]. Systemic targeted therapy [5] or immune checkpoint inhibitors [6] can also be started in selected patients with metastatic kidney cancer that cannot undergo cytoreductive nephrectomy. Additionally, patient satisfaction with this MDT approach has been proven to be excellent [7], which can ease disease-related anxiety and distress during this exceptional period. In conclusion, we believe that patient counselling and informed consent, accompanied by a traceable MDT approach, can provide an ideal setting for management of patients with cancer during this pandemic.
